# Effect evaluation of two types of dementia-specific case conferences in German nursing homes (FallDem) using a stepped-wedge design: study protocol for a randomized controlled trial

**DOI:** 10.1186/1745-6215-15-319

**Published:** 2014-08-12

**Authors:** Sven Reuther, Daniela Holle, Ines Buscher, Olga Dortmann, René Müller, Sabine Bartholomeyczik, Margareta Halek

**Affiliations:** German Center for Neurodegenerative Diseases (DZNE), Witten, Stockumer Str. 12, 58453 Witten, Germany; School of Nursing Science, Witten/Herdecke University, Stockumer Str. 12, 58453 Witten, Germany

**Keywords:** Dementia, challenging behavior, case conference, nursing home, stepped-wedge design, psychosocial intervention

## Abstract

**Background:**

Case conferences for people with dementia and challenging behaviors (e.g., apathy) are recommended as useful tools that enable staff in nursing homes to understand the behavior of people with this type of disease. Understanding peoples’ behaviors is the basis for the initiation of targeted interventions to improve the quality of care for people with dementia. Furthermore, case conferences demonstrate positive effects on burnout, dementia-specific burden, and vocational action competence of the staff. The two likely approaches for conducting case conferences include the following: A) using a structured assessment instrument, which guides the staff in understanding the residents’ behaviors and B) using a narrative approach in which the staff must identify the reasons for the residents’ behaviors in an unstructured manner. Case conferences are a complex intervention, and evaluating their multiple effects is challenging. The aim of this study protocol was to describe a likely solution for evaluating this type of complex intervention using a special cluster randomized trial.

**Methods:**

In this stepped-wedged cluster randomized trial, the two interventions will be sequentially implemented every three months in a group of 12 nursing homes (clusters) with a minimum of 360 residents over 19 months (7 months of intervention for each cluster and follow-up). The primary outcome is the reduction of challenging behavior (measured with the neuropsychiatric inventory-nursing home version [NPI-NH]). Secondary outcomes are residents’ quality of life, prescription of psychotropic medications, staff burnout, dementia-related stress, and vocational action competence. The effectiveness of the study will be accompanied by a process evaluation. The primary data will be analyzed using a Bayesian mixed effect model; the secondary data will be analyzed using descriptive statistics and mixed effects models.

**Discussion:**

The implementation and effect measurement of complex interventions such as case conferences within a cluster randomized trial are challenging (e.g., complex and intensive training, delayed treatment effect). In this study protocol, the methodological advantages and disadvantages of using the stepped wedge design to answer the research questions are discussed.

**Trial registration:**

http://www.controlled-trials.com/ISRCTN20203855; registered 10 July 2013.

## Background

People with dementia usually develop at least one challenging behavior in the course of the disease [1]. Challenging behavior influences both the quality of life of individuals with dementia and the caring situation of the caregivers [2]. The staff in nursing homes are confronted with demanding and complex care situations daily, which is associated with staff distress and burden [3]. Because pharmacological treatments yield only moderate benefits and adverse effects [4], safe and effective psychosocial interventions are needed to manage the challenging behavior of people with dementia [5]. Challenging behavior can be understood as a response to unmet needs. Therefore, the key prerequisite for person-centered management of challenging behavior [6] and initiation of individualized interventions [7, 8] is the search for causes of challenging behavior. However, understanding the underlying causes and triggers of challenging behavior is both demanding and complex and requires unique skills. Case conferences (CCs) provide opportunities for care teams to practice reflective communication, to engage in problem-solving with residents, to grow professionally, and to provide and receive emotional support for difficult work situations. The aim is to create a common understanding of a case. Although CCs are recommended for use in dementia care [9], there is still a lack of evidence to support their effectiveness [10]. The few studies that have been performed in the field of dementia have demonstrated that CCs reduce challenging behavior in people with dementia, positively influence vocational action competence, and reduce the staff’s work-related burden in nursing homes [11, 12]. However, due to the poor methodological quality of these studies, the results must be carefully interpreted. The studies highlight the need for methodologically well-designed intervention studies to provide more conclusive evidence of the effects of CCs on the care of people with dementia [10, 13].

Two different approaches to CCs are most commonly described in the literature, which differ primarily in the standardization of approaches for identifying the causes of challenging behavior in people with dementia [10]. One approach follows a standardized method of using assessment instruments and guidance in the diagnostic process [14, 15]. The other approach supports an open-thinking method for determining the potential causes of challenging behavior rather than relying on assessment instruments [11]. The effect of using a standardized assessment instrument to understand the process of challenging behavior is not clear, and the advantages of an open approach (without a standardized assessment) have not been explored in detail [10, 13]. Therefore, the aim of this cluster-randomized trial is to assess the effect of two different types of dementia-specific CCs, focusing on challenging behavior.

### The primary research question of the effectiveness study

The primary research question is: do the two different types of CCs have an effect on the prevalence of challenging behavior in people with dementia in nursing homes?

### The secondary questions

Secondary research questions include the following: do the two different types of CCs have an effect on 1) quality of life of people with dementia in nursing homes; 2) use of psychotropic medications for people with dementia in nursing homes; 3) burnout and work-related stress of the staff who care for people with dementia in nursing homes; and 5) vocational action competence of staff who care for people with dementia in nursing homes?

## Methods/design

### Study design

**Figure 1:**
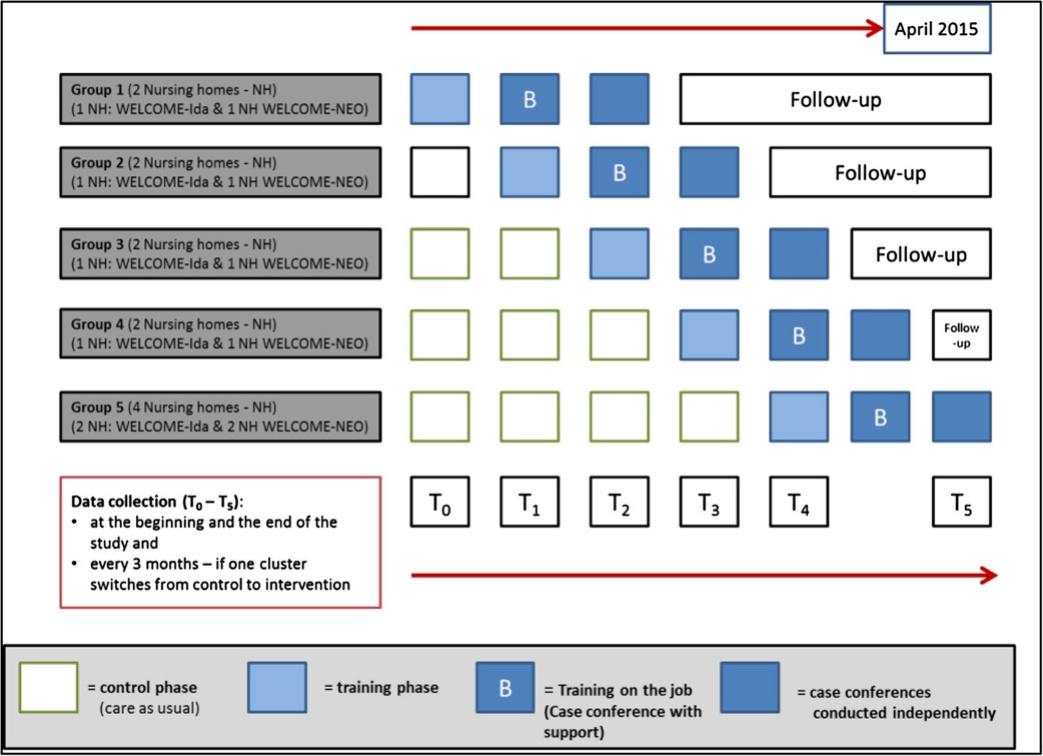
**Description of the stepped-wedge design of the dementia-specific case conferences in German nursing homes (FallDem) study.**

The cluster, randomized, controlled trial will be conducted using a stepped-wedge design (SWD) [16]. In this study, twelve different nursing homes (clusters) will participate in the study. Both interventions, (A) the Wittener model of CCs for people with dementia - the innovative dementia-oriented assessment tool (WELCOME-IdA) and (B) the Wittener model of CCs for people with dementia - the narrative approach (WELCOME-NEO), will be rolled out sequentially and in parallel every 3 months to two nursing homes (groups 1 to 4) over a period of 19 months, and the type of intervention will be randomly allocated (WELCOME-IdA will be allocated to six clusters and WELCOME-NEO will be allocated to six clusters). The last group (group 5) will contain four, instead of two, clusters because two clusters serve as reserves in case a cluster drops out during the intervention study (Figure 1). The primary intervention (training and performing CCs) takes 7 months, followed by a follow-up period that lasts until the end of the data collection in all of the clusters. Thus, each cluster will have the same length of intervention but different lengths of pre-intervention time and follow-up time.

### Sample size calculation based on the primary outcome

According to Hussey *et al.* (2007), the power calculation for a SWD depends on the number of clusters, number of steps, number of participants per cluster, strengths of the desired treatment effect, variability (expressed with the between-cluster variance *τ*^2^ and within-cluster variance *σ*^2^ components), and significance level α [17]. Due to the logistical, timing, and financial aspects of the study, we plan to recruit five nursing homes per intervention arm; we feel optimistic in obtaining informed consent to participate in our study from at least 30 residents per home (n = 150). Six time points in addition to baseline will be needed to ensure that every nursing home will receive the intervention. Based on the experience reported in an earlier study [18], a reduction in the prevalence of challenging behavior from 0.89 to 0.77 after the intervention WELCOME-IdA is assumed. Here, the prevalence rates will be calculated in the study based on the neuropsychiatric inventory-nursing home version (NPI-NH) (presence or absence (0/1) of at least one of the 12 challenging behaviors).

The between-cluster variance (*τ*^2^) and within-cluster variance (*σ*^2^) are estimated, according to Hussey *et al.* (2007) [17], as *σ*^2^ = 0.00326 and *τ*^2^ = 0.198. The significance level α is set at 0.05. The increase in the outcome variance due to the between-cluster heterogeneity is quantified by the inter-cluster coefficient of variation (*k*). Even if we assume *k* to be 0.5, rather than the maximum of usually observable inter-cluster coefficients of variation, under all assumptions mentioned above, the trial would achieve more than 80% power. The primary outcome is associated with the resident-level data. At this level, the study population consists of old and often multi-morbid individuals. Therefore, we should account for a minimum dropout rate of 20% during the study. Because of the flexible design, we will be able to adjust the sample size by substituting the dropouts with new residents with dementia. In case an entire cluster prematurely withdraws from the study, one additional home will be used as a reserve. Consequently, 180 residents from 6 nursing homes will be included in the study to test the effectiveness of WELCOME-IdA. The same dropout effects are expected for WELCOME-NEO. Therefore, we calculated using the same number of clusters and residents. In total, 360 residents from 12 nursing homes will participate in the study.

### Recruitment and randomization

Nursing homes are invited (by advertisement in a nursing journal) to apply for participation in the study. From this pool of interested nursing homes in North Rhine-Westphalia (NRW), 12 nursing homes will be randomly (simple randomization procedure) selected. Additionally, the type of intervention and when the cluster (nursing homes) switches from the control to the intervention protocol will be randomized. This process will guarantee that all 12 nursing homes have a similar likelihood of utilizing a particular type of intervention at the same time [19]. Then, the leaders of the nursing homes are responsible for the recruitment of the units and the residents according to the inclusion and exclusion criteria of the study. Here, all eligible participants of the participating units are invited to participate. Before the recruitment procedure will commence, each leader of the nursing homes will attend a kick-off meeting held by a senior investigator about the inclusion and exclusion criteria and the planned recruitment strategy. For the participants who drop out of the trial, we are planning to monitor the reasons (for example, death or moving) and perform a sensitive analysis at the end of the trial to determine whether they differ according to certain characteristics (for example, the prevalence of the challenging behavior or gender). Residents who are newly admitted to clusters during follow up will also be included in the study because we are interested (in a secondary analysis) in measuring the effect of the intervention on residents who move into the cluster.

### Methods to prevent contamination and selection bias

In the SWD, every nursing home acts as its own control group for the horizontal comparison of data. To avoid contamination of data in the vertical comparisons (between the clusters), the nursing homes will not be informed about the other participating nursing homes to prevent any type of communication and information exchange. Additionally, the data collectors and statistician will be blinded to the type of intervention (CC model) and the study phase (pre-intervention time).

### Interventions and control phases of the study

Both interventions (WELCOME-IdA and WELCOME-NEO) are defined as the reference interventions for each arm and therefore act as their own control groups (WELCOME-IdA versus control and WELCOME-NEO versus control) with respect to the stepped wedge design. In the control phase, in addition to primary and secondary data, the normal care practices (for example, existing care concepts, organizational aspects, and meeting forms similar to the CCs) will be assessed following the better reporting of interventions: template for intervention description and replication (TIDieR) guidelines [20] for each nursing home.

### The intervention

Both CC interventions (WELCOME-IdA and WELCOME-NEO), together with the key characteristics and the different process structures, were tested regarding their feasibility in two previous pilot studies [21, 22]. According to the results, both models were modified and adapted according to the current nursing home situation, were validated on behalf of the literature and expert interviews, and were pretested in one nursing home (manuscript in preparation). Both models have the same theoretical basis, key characteristics (group size, location, duration, sequences, number of participants and their functionality) (Table 1); however, the models differ in operationalization of the sequences with and without an assessment instrument (Table 2) [23].

**Table 1:** **Key characteristics of both case conference models**

**Group size**	5 to 8 individuals
**Location**	Undisturbed room
**Duration**	Estimated as 60 to 90 minutes
**Participants of the case conferences**	■ → Moderator of the case conference
	■ → Head of the ward or his/her assistant
	■ → Other staff working on the ward
**Role structure**	■ → Moderator
	■ → Time keeper
	■ → Case reporter
	■ → Reflecting partners

**Table 2:** **Key sequences of both models of case conferences and the differences between models**

	WELCOME-NEO	WELCOME-IDA
**Approach of understanding**	Process of understanding in a narrative approach (Neo), without any assessment instrument	Process of understanding using an assessment instrument (IdA) in case conferences
**Sequences**	**1. Preparation of the case conference**	
	**2. Introduction** (Welcoming, timeframe, roles)	
	**3. Description of the problem** Detailed narrative description of the case (what is the problem?)	**3. Description of the problem** Systematic description of the behavior using the questions of the assessment instrument (IdA)
	**4. Analysis of the situation** Finding possible reasons for the behavior within the team	**4. Analysis of the situation** Finding reasons for the behavior using the questions of the assessment instrument (IdA)
	**5. Planning of care interventions** (based on the analysis of the situation)	
	**6. Closing** (for example, personal reflection—what have I learned from the case?)	
	**7. Post-processing of the CC** (for example, responsibility to transfer the results into daily care routines)	

WELCOME-IdA: written model of case conferences for people with dementia - innovative dementia-oriented assessment tool; WELCOME-NEO: witten model of case conferences for people with dementia - narrative approach.

### Theoretical basis of both interventions

**Figure 2:**
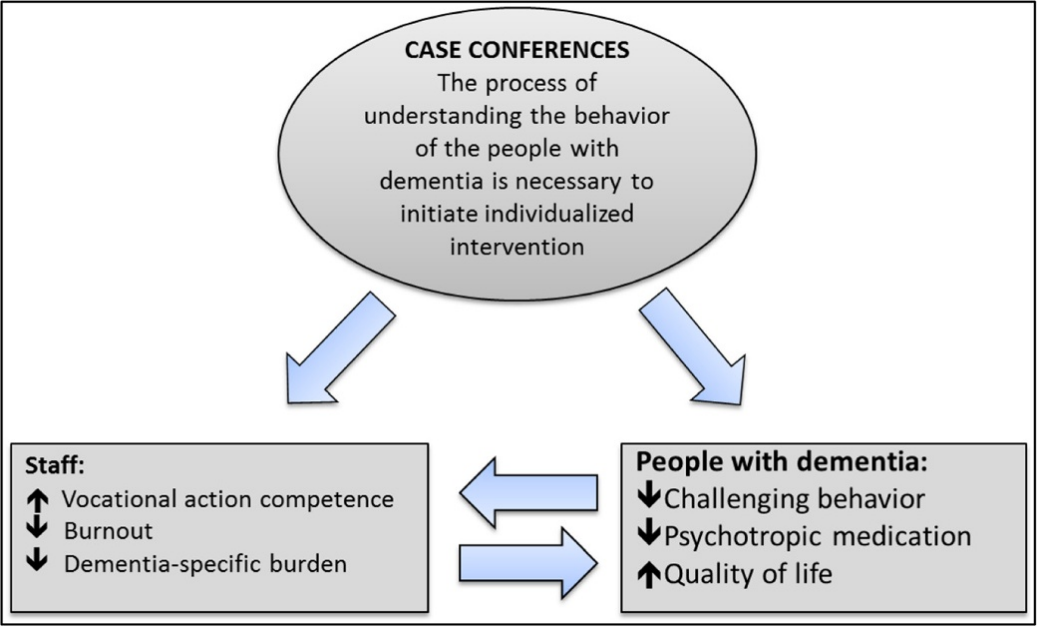
**Relationship between intervention and expected outcomes.**

Hermeneutics, which refers to the philosophy of understanding and the science of textual interpretation, has a two-fold objective: (a) orientation and the nature of understanding and (b) the interpretation of texts [19, 24] (Gadamer 1999; Ricoeur 1991). Defining hermeneutic principles as a core element of CCs is related to the ability to translate a particular situation to a universal context and to an overall interpretational framework [24]. The concept of attachment and the translation process are important, which Gadamer (2004) defines as authentic participation. Naden (2007) notes that this concept also refers to the aspect of consciousness-raising [25]. Understanding and interpretation are realized when reflection and contemplation occur, thus representing core principles of CC [26, 27], and are useful for understanding challenging behavior. All of the CC models used or known by the experts are also based on hermeneutic principles. Therefore, hermeneutics was chosen as a general theoretical framework for CCs [26, 27]. However, hermeneutics appears to be insufficient as the only theoretical background for CCs for residents with dementia because it disregards specific concepts associated with behavior in individuals with dementia. It may be assumed that care teams require a more specific theory for understanding the specific meaning of challenging behavior of residents with dementia [10, 23]. Thus, a dementia-specific theory (need-driven dementia-compromised behavior (NDB) model) for understanding challenging behavior and forming the content of our intervention was also selected [28]. In the NDB model, behavior is treated as a meaningful indicator of an unmet need. Therefore, dementia-related behavior can be explained as the staff’s inability to understand the needs of individuals with dementia and as the incapacity of people with dementia to express their own needs. Antecedents of NDBs include background factors and proximal factors. These factors can structure the search for causes and triggers of challenging behavior. In general, the understanding of these behaviors is the basis of initiation of individually tailored interventions, which could lead to a reduction in the prevalence of challenging behavior among residents, to an increased quality of life and to a reduction in the prescription of psychotropic drugs. Furthermore, it is expected that CCs result in an increase in the staff’s vocational action competence, reduced burnout, and decreased work-related stress (Figure 2).

In the intervention WELCOME-IdA, staff are guided systematically through the process of searching for causes and triggers of behaviors using the innovative dementia-oriented assessment tool (IdA). The practicability and validity of the instrument was tested in a previous study [29]. The IdA is a comprehensive assessment instrument based on the NDB model mentioned above and is utilized in a two-step process: the first step is a detailed description and quantification of the challenging behavior and the second step is the search for potential causes of the behavior. The second step of IdA is divided into five dimensions (state of health and independence in everyday life, communication, personality and life style before the onset of dementia, mood and emotions, and environmental influences) with specific guiding questions. The second intervention, WELCOME-NEO, uses no structured assessment. Understanding the situation of people with dementia requires the description and analysis of the behavior in an unstructured, free, narrative manner [21].

The intervention (components of the intervention) in both CC models will start with training in the respective CC model (WELCOME-IdA; WELCOME-NEO) and will be followed by four supported CCs (training on the job). Subsequently, a minimum of four CCs will be performed without any assistance (CCs without support).

### Educational strategy for the intervention groups

From previous studies [10], important components of specific CC implementation strategies were identified (Table 3) as follows: kick-off meetings with the staff, training in dementia and challenging behavior, training in moderation, CC reminders, a telephone hotline for queries, and the building of a steering group that is responsible for the development of a unit-specific implementation strategy. Three experts from an educational institution (Kaiserswerther-Diakonie) will conduct the training. The three experts are well-trained teachers with several years of experience in the field of CCs and in providing nursing care to people with dementia.

**Table 3:** **Components of the intervention and educational strategy for the implementation of interventions in both groups**

Components of intervention and implementation		Content	Participants	Duration
**Components of the intervention**	**I. Training in performing case conferences**	Aim and structure of CCs, NDB model, communication rules, use of IdA (only in the IdA intervention group)	Unit leaders, manager of the nursing home and members of care teams	Half a day
	**II. Case conferences with support** (training on the job)	Conducting CCs supported by the experts; the experts assist the moderator, are involved in the discussions, and provide advice	Members of care teams and experts	Minimum 2 case conferences within 3 months
	**III. Case conferences without support**	Conducting CC unaided	Members of care teams	Minimum 3 case conferences within 4 months
**Educational strategy for the implementation**	**I. Kick-off meetings**	Information concerning the project/intervention and data collection, time frame, organizational aspects	Participating care team members	90 minutes
	**II. Training in dementia and challenging behavior**	Diagnosis of dementia, forms and symptoms of dementia, causes of challenging behavior and management of challenging behavior	Members of care teams	Half a day
	**III. Training in moderation of case conferences**	Training in moderation techniques	2 nursing staff from each unit	2 days
	**IV. Training in development of implementation strategy for management**	A steering group will be formed to develop an implementation plan	Unit leaders, manager of the nursing home, moderators, project manager	2 days
	**V. Telephone-hotline and reminders**	Questions concerning the CCs and reminders via phone call to conduct CCs regularly	All participants	During the intervention

CC, case conference; NDB, need-driven dementia-compromised behavior; IdA, innovative dementia-oriented assessment tool.

### Measurements in residents with dementia

#### Primary outcome

The neuropsychiatric inventory-nursing home version (NPI-NH) [30], which is a common and validated instrument for detecting challenging behavior in elderly people with dementia [31], includes 12 subscales (delusions, hallucinations, agitation, depression, anxiety, euphoria, apathy, disinhibiting, irritability, aberrant motor behavior, night-time disturbances, and change in appetite) [31]. The staff members assess the frequency and severity for each domain. The severity index (frequency × severity) is calculated for each domain and for the overall scale. The scores range from 0 to 12 (for each domain) and from 0 to 144 for the total scores. The higher scores indicate the presence of more severe and challenging behaviors. The primary outcome will be coded as the presence or absence (0/1) of at least one challenging behavior measured using the NPI-NH.

### Secondary outcome

The quality of life instrument for dementia (QUALIDEM) [32], which was developed and validated in the Netherlands and was translated into and tested in German [33], consists of two consecutive versions for people with mild-to-severe dementia and those with very severe dementia. The version for mild-to-severe dementia assesses the following factors: care relationship, positive affect, negative affect, restless tense behavior, positive self-image, social relation, social isolation, feeling at home, and ability to stay occupied (37 items). The list contains 16 indicative items and 21 contra-indicative items that constitute nine homogeneous subscales. The second version for very severe dementia is based on the following factors: care relationship, positive affect, negative affect, restless tense behavior, social relation, and social isolation (18 items). For both versions, four response options are used: never, seldom, sometimes, and often. A higher score on a subscale indicates a higher quality of life.

The defined daily dose (DDD) of psychotropic drugs, which is a reliable instrument for measuring the volume of medications [34], is defined as the average maintenance dose of a medication when used according to its major indications in adults. The prescribed psychotropic agents will be obtained from the nursing records

### Measurements in nursing staff

#### Secondary outcomes

The Copenhagen burnout inventory (CBI), which is a widely used and validated instrument [35], focuses on exhaustion and measures burnout using three sub-dimensions: personal burnout (six items), work-related burnout (seven items), and client-related burnout (six-items). All of the items have five response categories (always, often, sometimes, seldom, never). The responses are rescaled to a 0 to 100 metric (with values of 0 to 25 to 50 to 75 to 100), and the total score of the scale is the average of the scores of the items. Individuals are defined as exposed to burnout with a score of >50 points [36].

### Work-related stress (*Bela-Dem*)

The dementia-specific stress on the staff will be assessed using the German *BelaDem* Questionnaire, which consists of 16 items that rate dementia-specific burden in relation to the challenging behavior of the residents. Each item will be rated by severity (0 = not at all, 1 = low, 2 = slightly, 3 = moderate, 4 = strong, 5 = very strong) and frequency (1 = never, 2 = rarely, 3 = sometimes, 4 = often) during the last four weeks. The scores will be calculated for each item as follows:


Each item ranges from 0 to 10, and a higher score indicates a greater stress level. The internal consistency and construct validity of the instrument has already been tested [18].

### Competence - reflection inventory (KRI)

The German questionnaire, the competence - reflection inventory (KRI), which will be used to assess the vocational action competence of the nursing staff, measures the dimensions vocational and methodological expertise, social competence, and self-competence (36 items). Each item is rated in steps of 10 from 0% (strongly disagree) to 100% (strongly agree) [37]. The scores are calculated by taking the mean of the items in each subscale; 50% indicates a normal level of vocational action competence, more than 50% indicates an above-average level of vocational action competence, and less than 50% indicates a low level of vocational action competence.

### Control variables

Several variables will be assessed at each time point, T (T0 to T5) as possible predictors of primary and secondary study outcomes measured at the resident, staff, and institutional levels. The functional assessment staging (FAST) score [38] will be used to classify the stage of dementia severity in this study. The activities of daily living will be measured using the Physical Self-Maintenance Scale (PSMS) [39]. Age, gender, and pain severity and frequency according to the study by Achterberg *et al.* [40] and the level of care are further covariates at the resident level. The demographic data, occupational skills, and work experience will be collected at the staff level.

### Institution-level measures

To evaluate several important structural and financial aspects of nursing homes and the potential institutional influences in relation to the characteristics of the respective unit, a self-developed questionnaire will be used. The questionnaire is divided into two domains. The first domain consists of four items and evaluates the entire institutional structure and financial aspects. The second domain focuses on the nursing unit and is divided into resident-specific factors (three items), structure (one item), and staffing, including staff turnover (five items). The single items of the questionnaire were previously tested with regard to practicability and feasibility in two studies [41, 42]. The questionnaire will be completed every 3 months by the study coordinator of the corresponding institution.

### The dementia milieu assessment (DMA)

The DMA assesses dementia-friendliness environments and consists of 29 items. The DMA is a standardized observation instrument that was used in a previous study [41]. The factors are divided into an environmental domain (21 items) and a psychosocial domain (8 items). Each item is measured using yes or no dichotomous-response options. The scores range from 0 to 21 for the environmental domain and from 0 to 8 for the psychosocial domain. A higher score indicates a more dementia-friendly environment. The DMA is used during a 2-hour period of observation from 3 to 5 pm in each facility. Observations will only be conducted in public spaces. A trained rater will complete the DMA before starting the intervention and after finishing the intervention.

### Process evaluation

The process evaluation aims to explore the delivery of the intervention and the implementation strategy to the clusters, the response of clusters and individuals to the intervention and the implementation strategy, the recruitment of clusters and individuals, as well as the context and contextual factors that promote or inhibit the implementation of the intervention (Figure 2). For the process evaluation, the framework for cluster randomized controlled trials (RCTs) [43] will be used. The details of the comprehensive process evaluation will be published separately.

### Inclusion criteria

#### Institution level

At least two units of one nursing home must participate in the study, from which at least 30 residents with dementia can be recruited. The care of the residents must predominantly take place in the respective unit.

#### Resident level

Criteria for inclusion are informed consent obtained from people with dementia or their legal representative; diagnosis of dementia based on the medical diagnosis in the charts and a FAST score > 1); residence for at least 14 days in the unit.

### Staff level

All of the nursing staff working in one of the two participating wards of the nursing home must provide their informed consent.

### Exclusion criteria

#### Institution level

Excluded will be nursing homes with <30 residents with dementia; homes with reconstruction work ongoing in the participating unit; homes participating in any other research project at the same time, which requires personal resources.

#### Resident level

Exclusions will be patients with schizophrenia or any other type of diagnosed psychiatric disease (found in the nursing documentation); residents with dementia in day or night care who are outside the unit for most of the time.

#### Staff level

Nurses who are not working permanently in one of the two participating units of the nursing home or are employed temporarily will be excluded.

### Data collection and measurements

The data collection to assess the primary and secondary outcomes will be conducted every 3 months over a period of 17 months (Figure 1). The maximum intervention period for each cluster varies according to when the nursing home switches from the control phase to the intervention phase, which occurs between 7 and 17 months. The measurement instruments were chosen based on their appropriateness for the target setting and population, psychometric properties (validity and reliability), and their feasibility. The data at the resident level (for example, health indicators, behavior) will be obtained from interviews using the caregivers as proxies. The trained study assistants will simultaneously interview two caregivers who know the resident very well. If there is no agreement between the caregivers, the response of the caregiver with the longest relationship with the resident will be chosen. Every study assistant rater (primarily students) will undergo a two-day training session on the use of the questionnaires and instruments and will receive a detailed manual for the data collection, questionnaires, and instruments. For the first data collection session, the raters will be assisted by a senior researcher and a junior researcher to ensure that the data are collected as planned. The staff will complete questionnaires at the institutional and staff levels. The questionnaires will be tested to assess their feasibility in a pretest in one nursing home before commencing the intervention study.

### Ethics and dissemination

The Institutional Review Board for Ethics in Research, German Society for Science in Nursing (E-DG-P) has discussed and considered the proposal *Fallbesprechungen bei Menschen mit Demenz (FallDem), Teil II: Interventionsdurchführung* (delivered in August 2011) and imparts an ethical clearing. Informed consent from each participant (residents and staff) will be obtained before the start of the trial. We will publish the main outcomes of the study in peer-reviewed scientific journals and journals for nursing practice and present the results at national and international conferences. Furthermore, based on the key results, we plan to write a handbook for nursing homes on how to use and implement CCs into their daily work routines.

### Analysis of data for effectiveness

In general, the analysis will follow the principles of an intention-to-treat analysis at the cluster level. The missing data will be appropriately imputed after assessing the missing data mechanism. For the analysis of the secondary staff’s outcomes, the missing data will be censored regardless of the reason for missing data.

### Primary data analysis

The statistical analysis will be conducted after the end of the follow-up phase (T6). For the main analysis of the primary outcome, differences in the NPI scores of the clusters within the two intervention groups (WELCOME-IdA and WELCOME-NEO) for the different time points will be analyzed. Here, the NPI-NH scores of the control condition will be compared with the data from the intervention phase within each intervention arm [44]. With respect to the nature of the SWD, the clustering, repeated measurements, and confounding effect of time must also be considered in the data analysis. With respect to our binary outcome, the use of a generalized linear mixed-effects model is highly recommended for data obtained from such types of studies. These models allow the inclusion of both fixed (for example, intervention, time) and random (for example, nursing home, resident-observed several times) factors [17]. Furthermore, a delayed intervention effect of the CCs is assumed because the nurses need time to implement the procedure. Thus, the duration of the intervention in months must be considered. For the estimation of model parameters, the Bayesian approach will be used. In contrast to the likelihood-based inference, the Bayesian approach is more flexible and powerful because it allows, for example, the inclusion of prior information from previous studies. For the model selection process, an approach will be used that was previously described by Cheng *et al.* [45]. If necessary, the influence of missing data will also be analyzed by sensitivity analyses.

### Secondary data analysis

The secondary outcome parameters will be analyzed using descriptive statistics and mixed effects models to generate hypotheses for additional research (for example, reduction of staff burden, differences between the two concepts of CCs).

## Discussion

The effectiveness of CCs on challenging behavior, quality of life of people with dementia, prescription of psychotropic drugs, nursing staff’s burnout, dementia-specific burden, and vocational action competences will be evaluated. The use of the SWD offers several methodological and logistical advantages over the traditional randomized cluster design. RCTs are the gold standard for measuring clinical effects. In some situations, it is not possible or it is unethical or unfeasible to deny an intervention to an organization, a team, or residents. CCs are widely used in nursing homes and are recommended in guidelines [9]. The traditional waiting-list designs seem to overestimate the effect of interventions [46]. Therefore, alternative approaches for evaluating clinical and community interventions have received increased attention, particularly those that can retain several elements of randomization and that can be considered as controlled trials [16]. The SWD is described in the literature as an alternative approach because it avoids several methodological pitfalls associated with before and after designs and it retains controlled data elements and randomization [47]. Cluster-randomized trials are used to test whether an intervention is replicable and recognizable. However, CCs represent an example of a complex intervention in which the framework may be standardized but not each individual component. The way in which staff conduct CCs cannot be completely controlled because there is also a type of ongoing group dynamic process, which could change from case to case; often, the process must change because of the specifics of the case. For complex interventions, it is more important to standardize the process and the function of the intervention [48] rather than all of the individual components of the intervention. In this study, we describe key obligatory elements of the intervention. With the long follow-up period of the trial, we will be able to detect intervention changes over a long time period.

Several logistical advantages of the study design exist. The staggered introduction of the intervention allows time to provide training on CCs and supports the stepwise study design. If we chose to implement a traditional cluster randomized trial, we would need to start with the intervention at the beginning of the trial, which would not be feasible with respect to the resources of our research team. However, from the statistical point of view, the data analysis is complex (for example, control for temporal trends in outcome variables or accounting for repeated measurements) [47]. Only a few papers discuss the analysis methods in an SWD [17, 49, 50]. Additionally, some nursing homes will wait a long time to begin the intervention, which poses a potential risk that several nursing homes may drop out of the trial due to loss of motivation or organizational developments. This situation also affects the resident and staff samples, and the problem of missing data intensifies.

Measuring the effect of psychosocial interventions in health care research is, for the most part, a challenge because of their complexity [51]. Complex interventions often consist of different components that may contribute to the success or failure of the effect of an intervention. Often, the environment in which the intervention will be implemented is also complex [52]. RCTs are stated to be the most powerful design for proving the effect of an intervention; however, these trials are limited in their ability to explain the reasons why an intervention is effective [53, 54]. Therefore, it is important that an effectiveness study is accompanied by a process evaluation that provides additional information regarding the change processes and the contribution of each component of the intervention to the overall effect of the intervention. Otherwise, it will not be possible to gain insight into the so-called *black box* of this intervention [55].

### Limitations of the study

First, because of limited resources, the study will be conducted in only one state, North Rhine - Westphalia (NRW) in Germany, which must be considered in the generalization of the results (external validity). The funding of long-term care by the local governments could differ between states in Germany. However, NRW is one of the largest and most densely populated states in Germany. Nonetheless, the participating nursing homes can be compared with all of the nursing homes in NRW and in Germany with respect to how representative they are.

The second limitation is the small number of nursing homes (six for each intervention arm) and the simple randomization procedure, which could lead to an imbalance between the different nursing homes in relation to several important covariates. Several studies solve this problem by using a SWD with the constrained randomization procedure after obtaining baseline data [56, 57]. They balanced the allocation with respect to important covariates at the clinic level (for example, geographic region or prevalence rates of important covariates) [50]. However, this type of restricted randomization procedure is criticized even with the use of a small number of clusters because of their influence on the data analysis [58]. Additionally, in our case, we would need baseline data to restrain the allocation sequence to important factors, which seems unrealistic because of logistical reasons. Because we are unable to conduct the randomization procedure after baseline data collection, possible covariates were mentioned in the data analysis section. The third limitation is a possible selection bias at the unit and resident level. In our stepped-wedge cluster randomized trial, we randomize the nursing homes according to the type of intervention and the time at which the cluster switched from control to intervention phase. However, the selection of the unit and the residents based on the inclusion and exclusion criteria must be performed by the nursing home leaders because of logistical reasons. This selection strategy may increase the possibility of post-randomization selection bias at unit and/or resident level (for example, only very motivated units take part in the study or residents with high-intensity or low-intensity behavioral and psychological symptoms of dementia (BPSD) are chosen). To identify a possible selection bias due to the recruitment process, we will collect organizational and structural baseline data at the nursing home, unit and resident level. Additionally, the risk at the resident level is negligible because all eligible participants of the two units are invited [59]. However, at the end of the trial, we will compare the demographic data (for example, care dependency, age, sex) at the resident and unit level with data from the German Office of Statistics.

The fourth limitation concerns the data quality. We will use proxy instruments for the primary and secondary outcomes. We must consider that the challenging behavior assessed using the NPI-NH may be biased. As a proxy-rating instrument, the NPI-NH assesses the experience and subjective perspective of a professional caregiver of an individual with a particular behavior [60]. Another bias may occur if the staff believes they need to rate their own quality of care instead of the residents’ quality of life. To resolve this problem and to increase the objectivity, two staff members will generally be interviewed about people with dementia.

## Trial status

This trial was initiated in 2011 (development of the intervention) and will be completed by the end of 2015. The recruitment of the nursing homes was completed in fall 2013. The recruitment of the participating staff and residents (who will provide intervention training) will be completed by the end of 2014. Participating staff (who will provide intervention training) will be completed by the end of 2014.

## Abbreviations

BPSD: behavioral and psychological symptoms of dementia
Bela-Dem: work-related stress (*Belastungen bei Demenz*)
CBI: Copenhagen burnout inventory
CCs: case conferences
DDD: defined daily dose (of psychotropic drugs)
DMA: dementia milieu assessment
E-DG-P: Institutional Review Board for Ethics in Research German Society for Science in Nursing
FallDem: dementia-specific case conferences in German nursing homes
FAST: Functional Assessment Staging Tool
KRI: German questionnaire ‘competence - reflection inventoryʼ
NDB: need-driven dementia-compromised behavior
NPI-NH: Neuropsychiatric inventory-nursing home version
NRW: North Rhine - Westphalia
PSMS: physical self-maintenance scale
QUALIDEM: quality of life instrument for dementia
RCT: randomized controlled trial
SWD: stepped-wedge design
WELCOME-IdA: Wittener model of case conferences for people with dementia - innovative dementia-oriented assessment tool
WELCOME-NEO: Wittener model of case conferences for people with dementia - narrative approach.
